# Effect of ActiGraph’s low frequency extension for estimating steps and physical activity intensity

**DOI:** 10.1371/journal.pone.0188242

**Published:** 2017-11-20

**Authors:** Yuri Feito, Lyndsey M. Hornbuckle, Lauren A. Reid, Scott E. Crouter

**Affiliations:** 1 Department of Exercise Science & Sport Management, Kennesaw State University, Kennesaw, GA, United States of America; 2 Department of Kinesiology, Recreation, & Sport Studies, The University of Tennessee–Knoxville, Knoxville, TN, United States of America; 3 Department of Epidemiology and Biostatistics, University of South Carolina, Columbia, SC, United States of America; Universidad Europea de Madrid, SPAIN

## Abstract

This study examined the effects of the ActiGraph’s (AG) low-frequency extension (LFE) filter on steps and physical activity classification in the free-living environment. Thirty-four African-American women (age, 24.5±5.2 years; BMI, 24.9±4.5 kg/m^2^) had daily activity measured simultaneously with an AG-GT3X^+^ accelerometer and a New Lifestyles NL-800 pedometer for seven days. Steps per day (steps/day) and time (minutes/day) spent in sedentary, light, and moderate-to-vigorous physical activity (MVPA) were examined with and without the LFE filter (AG-LFE and AG-N, respectively). The AG-LFE recorded more total steps (13,723±4,983 steps/day) compared to AG-N and NL-800 (6,172±2,838 and 5,817±3,037 steps/day, respectively; *p*<0.001). Compared to the AG-N, the AG-LFE estimated less time in sedentary behaviors (518.7±92.1 vs. 504.2±105.4 min/day, respectively; *p*<0.001), and more time in light (247.7±70.4 vs. 279.1±74.7 min/day, respectively; *p*<0.001) and MVPA (18.9±16.9 vs. 21.5±18.2 min/day, respectively; *p*<0.001), respectively. These data suggest that steps and physical activity classifications will be affected when using the ActiGraph with and without the LFE filter. Future research should investigate the accuracy of these measures using the LFE filter.

## Introduction

Accelerometry-based activity monitors are widely used in research for the measurement of physical activity. ActiGraph™ (AG) devices are some of the most common devices used for research purposes, and have been shown to provide accurate estimates of physical activity in the free-living environment [1]. In 2009, AG introduced the low frequency extension (LFE) filter, which increased the device’s sensitivity to lower intensity activities; thereby, allowing for the measurement of a greater range of physical activity intensities [2]. In turn, this would hypothetically increase the accuracy of measuring physical activity in participants in the free-living environment, who may engage in lower intensity activities throughout the day.

Studies using objective measurements of physical activity have shown that African-American women of various adult ages are categorized as sedentary and low active per proposed step indices [3, 4], and spend more time participating in low-intensity physical activities compared to those of moderate and vigorous intensities [5, 6]. Data from the National Health and Nutrition Examination Survey (NHANES) have shown that non-Hispanic black females ages 20–59 years are next to last among adults in the United States for time spent in moderate and vigorous physical activity [7], with the lowest group (non-Hispanic white women) obtaining only 0.3 less minutes per day. As the premise behind the use of the AG’s LFE filter is to increase the sensitivity to capture lower-intensity activities, studying and implementing the use of the LFE may be warranted among specific groups shown to acquire mostly low-intensity activity (such as African-American women) in order to provide the most accurate measurements for these populations.

Recently, Feito and colleagues [8] examined the effects of using the AG with and without the LFE in a group of healthy men and women, and showed that the use of the AG-LFE provided significantly higher estimates of daily steps taken in the free-living environment, compared to the StepWatch™ activity monitor. These findings align with previous studies, which have demonstrated similar effects of the LFE when compared to older AG activity monitors (i.e. AG 7164) [9, 10]. However, neither of these studies used the AG monitor to examine activity patterns in a population often shown to participate in mostly low-intensity activity (e.g., African-American women), and only one examined differences in both steps per day and time spent in sedentary behaviors and each activity intensity [9]. Thus, the purpose of the current study was to examine the influence of the AG with and without the LFE on steps per day and physical activity intensity classification in a sample of low active African-American women.

## Materials and methods

### Participants

Participants of the current study were enrolled in a larger cross-sectional study examining the relationship between daily physical activity intensity, cardiorespiratory fitness, and metabolic risk between May and August of 2013. Forty-one apparently healthy, self-identified African-American women, age 19–40 years, participated. Recruitment strategies included posting flyers around the University and at various public venues in the surrounding community, as well as word of mouth. In addition, research staff visited meetings of local undergraduate and graduate chapters of several social organizations that targeted the study population, and distributed flyers electronically to these groups. Participants were excluded for any physical illnesses (cardiovascular or pulmonary disease, uncontrolled hypertension, or diabetes), pregnancy, smoking in the past six-months, or orthopedic disability that would limit their ambulatory function. In an effort to capture the most typical physical activity measurements for each individual, participants were not permitted to enroll in the study if they were currently participating in any type of exercise or weight loss program that was adopted less than three months prior to entry into the study. Forty-nine women contacted study staff to express interest in the study and 48 agreed to be screened for eligibility (S1 File). Of the 48 screened, participants were excluded for current/recent participation in a new exercise or weight loss program (n = 6) and recent surgery that limited physical activity (n = 1). For the present study, participants were also excluded due to monitor failure or not meeting the minimum wear-time criteria (n = 7).

Participants reported to the exercise physiology laboratory for orientation to the study and to undergo physical measurements. Before any study procedures took place, all participants read and signed an informed consent form stating the nature of the study. The informed consent form and all procedures were reviewed and approved by the University’s Institutional Review Board.

### Anthropometric measurements

Body weight (kg) and height (m) were measured using a Tanita^®^ digital beam scale with height rod (Tanita Corporation of America, Inc.; Arlington Heights, IL) in light clothing (e.g. shorts and t-shirt) and without shoes. Body mass index (BMI) was calculated from the measured weight and height.

### Physical activity monitors

AG-GT3X+ accelerometers (ActiGraph; Pensacola, FL) and NL-800 pedometers (New-Lifestyles, Inc.; Lees Summit, MO) were used to measure physical activity for seven consecutive days. Briefly, the AG-GT3X+ uses a Microelectro-Mechanical-System (MEMS) accelerometer to measure changes in acceleration in three individual orthogonal planes (vertical, antero-posterior, and medio-lateral), and also provides the vector magnitude [11, 12]. The NL-800 uses a piezoelectric accelerometer to measure steps, which has been shown to be more accurate compared to pedometers that use a spring-levered mechanism, particularly at slower walking speeds and in those with increased abdominal fat [13]. Additional information regarding these two devices can be found elsewhere [12, 14].

Both the AG-GT3X+ and the NL-800 were distributed during the first laboratory visit. An investigator placed both devices onto a belt that was worn around the participants’ waist at hip level; with the AG positioned on the right side of the belt at the midline of the thigh and the pedometer positioned on the left side aligned with the midline of the thigh. Although the placement of the AG varied slightly from manufacturer instructions (anterior axillary line), step counts have been shown to be consistent and accurate between both of these two placement sites [15]. To ensure the NL-800 pedometer was recording steps accurately, each participant completed a 20-step test around the laboratory while a researcher counted steps manually. In the event the NL-800 did not record steps accurately (within one step), monitor placement was altered or a new monitor was given to the participant and tested before the device was sealed to blind participants to step counts during the monitoring period. Unlike the NL-800, the AG-GT3X+ does not display any data for the wearer.

Participants were then given specific instructions to wear the belt with monitors attached for seven consecutive days during all waking hours, except during water activities. During the seven-day assessment period, participants were instructed not to alter their typical daily activities. Participants were also given an activity log on which they were instructed to record a summary of their daily activities and the time at which they put on and removed the belt each day. At the end of the seven-day monitoring period, a study investigator collected the activity monitors and activity logs.

### Statistical analyses

The AG’s raw data were collected at 60 Hz and uploaded using the ActiLife software (version 6.0). Raw files were converted to counts per minute in two different files using the normal (AG-N) and LFE (AG-LFE) filter. Wear time was examined using the procedures of Troiano et al. [7]. A valid day was defined as a minimum of 10 hours of wear time and each participant needed a minimum of four valid days to be included in the final analysis. As we were interested in comparing how the filtering mechanism affects the counts and physical activity classifications of the two filtering options, we used commonly used cut-points. However, it is worth noting that these cut-points were designed using data from the AG-7164, as there are no specific cut-points exclusively designed for the AG-LFE. As such, we used the cut-points developed by Matthews et al. [16], and Troiano et al. [7] for the AG-N and AG-LFE to determine minutes spent in sedentary behaviors (< 100 counts/min), and light (100–2,019 counts/min), moderate (2,020–5,998 counts/min), and vigorous (≥ 5,999 counts/min) physical activity. Due to the low amount of time spent in vigorous physical activity (average of 1.6 ± 3.8 minutes/day for the sample), moderate and vigorous physical activity were combined into one variable (MVPA). AG-measured counts and time spent per day in sedentary behaviors, light physical activity, and MVPA were then averaged across all valid days for each participant. NL-800 pedometer steps were retrieved and recorded as average steps/day.

Pearson product moment correlation coefficients were used to examine the nature of the association between the two AG device filters (AG-LFE and AG-N) and the NL-800 for steps/day. Differences between devices and filtering conditions (AG-LFE x AG-N x NL-800) were examined using repeated measures ANOVA for steps/day. In addition, a 2 x 3 repeated measures ANOVA was used to determine differences between the AG devices and each physical activity intensity category (sedentary, light physical activity, and MVPA). Additionally, we completed pair-sample t-test to explain the differences between the two-device filtering mechanism (AG-N and AG-FLE). Considering the small sample in this study, we calculated and report Eta-square (η^2^) to determine the strength of association between the device pairs. Finally, Bland–Altman plots [17] were constructed to show the variability of the devices’ error scores. With this technique, the mean error score and the 95% prediction intervals can be examined in graphical form. Devices that are in closer agreement will have a mean bias close to zero and tighter 95% prediction intervals.

All statistical analyses were conducted with SPSS version 22 for Mac (SPSS, Inc.; Chicago, IL). The alpha level was set at 0.05. All data are presented as mean ± standard deviation. S1 File provides descriptive and activity monitor data for all study participants.

## Results

Of the 41 women enrolled in the study, 34 were included in the final analyses (17% attrition). Seven individual files were eliminated due to monitor failure (n = 2) or not meeting the wear time criteria (n = 5). Participant demographic characteristics are presented in Table 1.

**Table 1:** Demographic characteristics of the study participants (N = 34).

	Age (years)	Height (m)	Weight (kg)	BMI (kg/m^2^)
Mean (± SD)	24.5 ± 5.2	1.7 ± 0.1	68.7 ± 14.6	24.9 ± 4.5

SD = Standard deviation.

**Fig 1:**
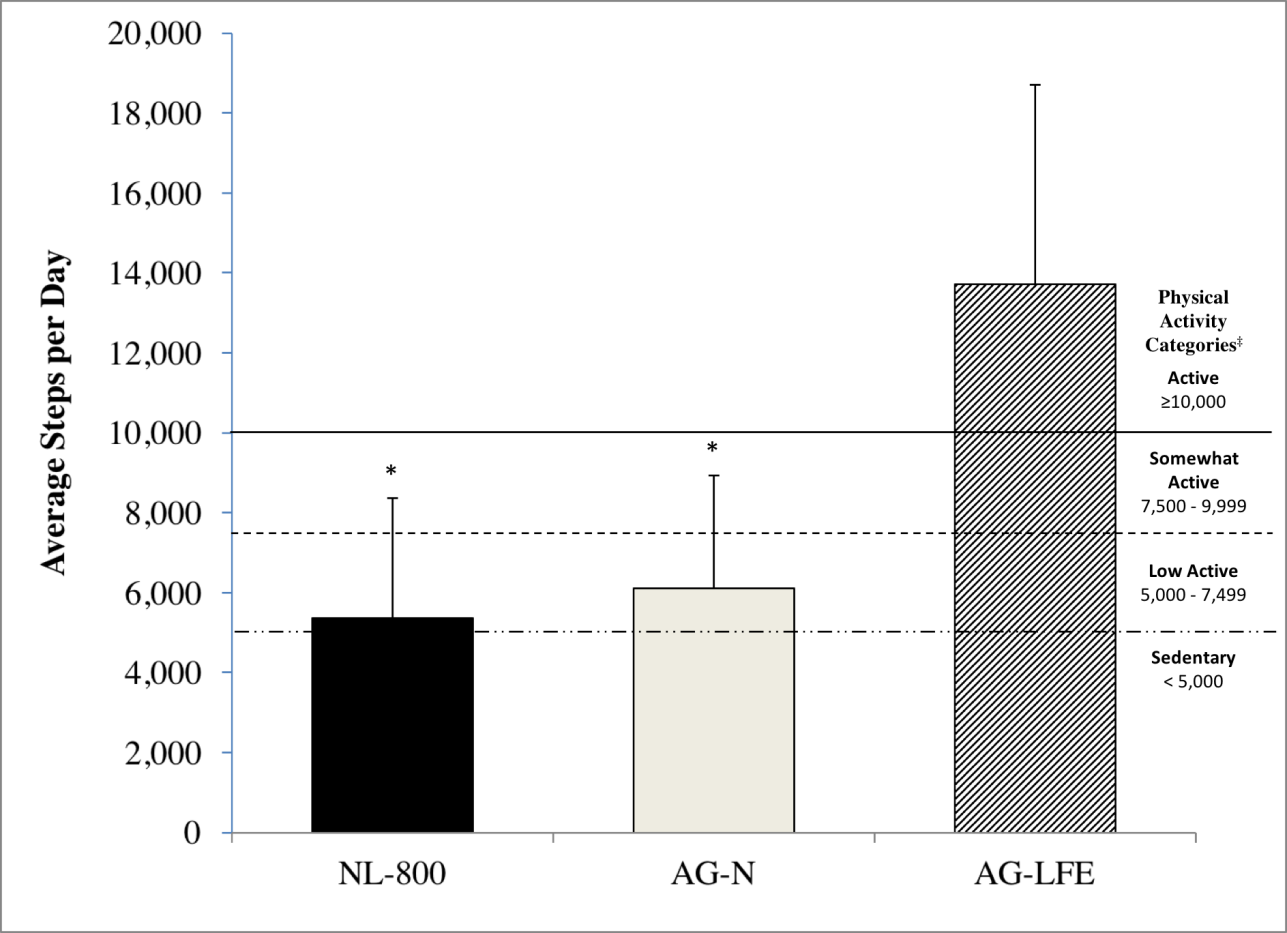
Estimated steps/day and activity level for each device. Average estimated steps/day and activity level categorization for the New-Lifestyles pedometer (NL-800) and ActiGraph GT3X+ accelerometer with (AG-LFE) and without (AG-N) the low frequency extension turned on (N = 34). Error bars represent the standard deviation. *Significantly different from AG-LFE (*p* < 0.001). ‡ Physical activity categorizations (steps/day) based on proposed indices for public health [18].

There were significant positive correlations between the AG-LFE and AG-N steps/day (r = 0.71; p < 0.001), the AG-LFE and NL-800 steps/day (r = 0.68; *p* < 0.001), and the AG-N and the NL-800 steps/day (r = 0.93; p < 0.001). Significant differences were observed among the three devices for steps/day (F (2) = 77.01, η^2^ = 0.828, *p* < 0.001) (Fig 1). On average, the AG-LFE estimated 13,723 ± 4,983 steps/day, which was 22% and 36% more steps/day than the AG-N and NL-800 (6,172 ± 2,838 and 5,817 ± 3,037 steps/day, respectively; *p* < 0.001). There was no statistical difference between the NL-800 and AG-N for steps/day (*p* = 0.197).

**Fig 2:**
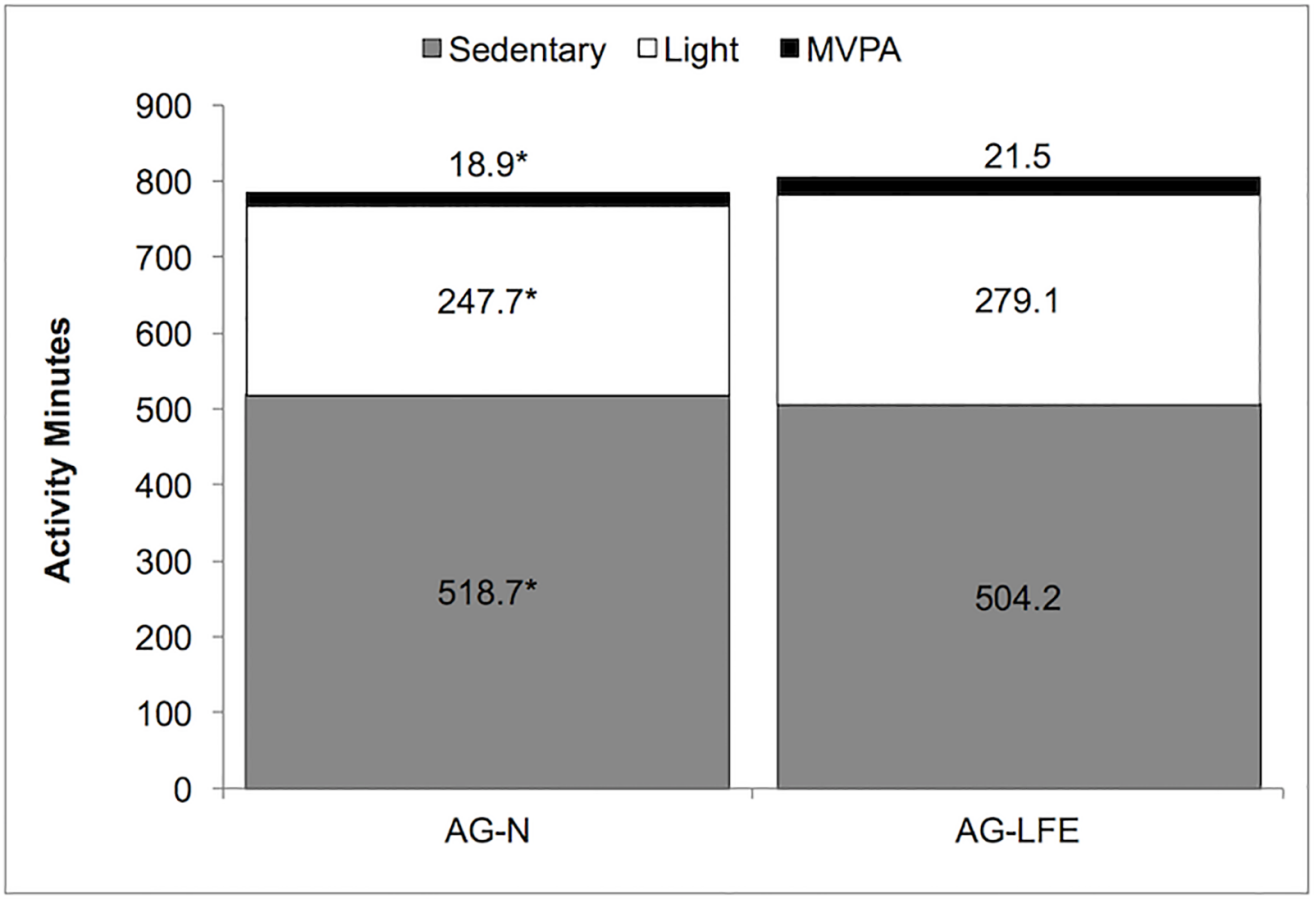
Mean time spent in each physical activity category per day. Mean time spent in each physical activity category per day for the ActiGraph GT3X+ accelerometer with (AG-LFE) and without (AG-N) the low frequency extension turned on (N = 34). *Significantly different from AG-LFE (*p* < 0.001). MVPA: moderate and vigorous physical activity.

Similarly, significant differences were observed between the AG-N and AG-LFE filtering options when estimating wear time (F(2) = 32.82, η^2^ = 0.672, *p* < 0.001). Overall, significantly more wear time was estimated when using the AG-LFE compared to the AG-N condition (804.7 ± 97.67 vs. 785.30 ± 83.68 min/day, respectively; F(1) = 11.09, η^2^ = 0.252, *p* = 0.002). On average, compared to the AG-N, the AG-LFE estimated significantly less time spent in sedentary behaviors (518.7 ± 92.1 vs. 504.2 ± 105.4 min/day, respectively; *p* < 0.001), and significantly more time spent in light (247.7 ± 70.4 vs. 279.1 ± 74.7 min/day, respectively; *p* < 0.001) and MVPA (18.9 ± 16.9 vs. 21.5 ± 18.2 min/day, respectively; *p* < 0.001) (Fig 2).

**Fig 3:**
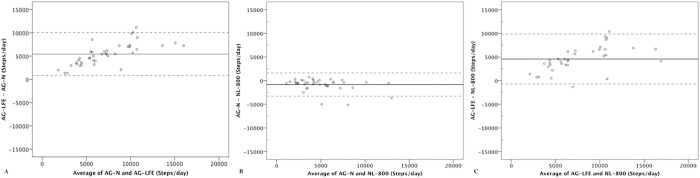
Bland–Altman plots comparing mean steps/day across a seven-day free-living measurement. A) ActiGraph with low frequency extension (AG-LFE) and ActiGraph with normal filter (AG-N), B) AG-N and New-Lifestyles pedometer (NL-800), and C) AG-LFE and NL-800. Solid lines represent mean bias; dashed lines represent 95% prediction intervals.

Fig 3 depicts difference scores through Bland-Altman plots. The AG-N and NL-800 had the closest agreement with a mean bias of -842 steps/d and 95% prediction intervals ranging from -3306 to 1622 steps/d. For the comparison of the AG-LFE between the AG-N and NL-800, the mean biases were approximately 5,000 steps/day for both with 95% prediction intervals ranging from -10,000 to 10,000 steps/day.

## Discussion

The purpose of this study was to examine the effects of the AG activity monitor LFE filter in a group of low active African-American women in a free-living environment. To our knowledge, this is the first study that specifically assesses the effect of this filtering mechanism in this population. Similar to previous studies [8, 19, 20], the findings from the current study suggest that the use of the AG-LFE filter significantly estimates higher steps/d compared to the AG-N filter and a commonly used pedometer (NL-800). These findings, along with those of Feito et al. [8], Tudor-Locke et al. [19] and Wallen and colleagues [20], suggests that the higher counts in steps/d may be related to the LFE filter rather than to the specific population being studied.

In 2013, Cain et al. [9] showed similar results as those presented here. In that study, Cain and colleagues reported discrepancies in steps/day among the AG with and without the LFE when compared to the AG-7164, with the AG-N recording 2,041 fewer steps/day, while the AG-LFE recorded 3,597 more steps/day. In addition, Wallen and colleagues [20] compared the AG’s normal and LFE filters during free-living conditions in individuals with Parkinson’s disease and showed significantly greater values for steps/day with the LFE filter compared to the normal filter (11,117 ± 4,553 vs. 4,730 ± 3,210 steps/day, respectively). More recently, Feito and colleagues [8] compared two models of the AG monitor (GT1M and GT3X) with the normal and LFE filters during treadmill walking and reported small differences (< 5%) between directly observed steps and estimated steps; however, the AG GT3X (with LFE) was most accurate (≤ 3%) at speeds above 54 m/minute. Even though using the LFE resulted in greater accuracy during treadmill walking, the increased sensitivity provided by the LFE filter resulted in step measurements that were over 30% higher than steps recorded by the StepWatch in a free-living environment. In this regard, however, it is worth noting that the StepWatch has recently shown limited accuracy for activities above 134 m/min [21]. Thus, it is possible that this overestimation reported by Feito and colleagues in the free-living environment may be related to the activities the participants were engaged in throughout the day (e.g. jogging, running, etc.) and not the device. Tudor-Locke and colleagues [19] also compared step outputs of the AG with the normal and LFE filters under free-living conditions and reported a doubling of steps/day recorded when using the LFE filter compared to the normal filter (13,029 vs. 6,743, respectively). In contrast, Webber and St. John [22] compared step counts of the AG-GT3X+ with the LFE filter and the StepWatch device and showed the step counts were similar for the two devices when placed on the ankles of hospitalized older adults who have slower walking speeds. Considering the varying results provided herein, this suggests that further validation studies comparing the AG’s LFE to the normal filter are warranted.

Based on the results of the current study, using the AG-LFE mean steps/d categorizes the participants as being somewhat active to active, while the use of the AG-N and NL-800 classifies them as low active [18]. Thus, the current results show that even though the AG has been deemed a reliable and valid device to measure steps in the free-living environment, at this time, it is unclear which step value is accurate. In addition, there is a need for validation studies in free-living environments that use criterion measures, such as video recording to hand tally the actual steps taken. Although this may seem problematic for investigators attempting to prescribe absolute levels of objectively measured exercise/physical activity, these devices are likely still appropriate to measure physical activity changes, regardless of the accuracy shown between models. Furthermore, researchers should be cautioned in applying the step guidelines to devices other than spring-levered pedometers, as they were developed using the spring-levered Digi-Walker pedometer. The Digi-Walker pedometer has been shown to under-count steps at slower walking speeds [23] and in overweight and obese individuals [13]; thus, application of these step guidelines to devices not influenced by walking speed or obesity would potentially give the appearance of being more active. Therefore, there is a need for step guidelines to be developed for use with newer generation devices that use accelerometer mechanisms.

A secondary purpose of our study was to examine how the LFE affects an individual’s physical activity intensity classification. In this group of women, the AG-LFE estimated less time spent in sedentary behaviors compared to the AG-N. In contrast, the AG-LFE estimated more time in light and MVPA than the AG-N. This is in agreement with findings previous findings from Cain and colleagues [9], who also showed significant differences in sedentary time and time spent in various physical activity categories between GT3X+ filtering mechanisms, where the LFE recorded less sedentary time, but more minutes of light, moderate, and vigorous activity than the AG-N. In addition, they also demonstrated that the use of the LFE provided more comparable results to the AG 7164, which has been used to develop the majority of cut-points and algorithms currently used with the AG. Therefore, these findings are again important to document, as they provide insight as to how the choice to use the GT3X+ with or without the LFE will impact physical activity classifications.

The current study shows that activating the AG’s LFE filter may generate different measurements of time spent in sedentary behaviors, light activity, and/or MVPA among African-American women. These findings coincide with those of Ried-Larsen et al. in 2012, which showed that activation of the LFE filter attenuated differences in time spent in sedentary and light physical activity compared to the normal filter, but significantly (*p* < 0.05) increased the time spent in moderate and vigorous physical activity [12]. Based on our findings we are unable to determine which filter option may be most accurate to estimate physical activity levels in the free-living environment. Even though previous investigations [8, 9, 12, 24] have shown differences between the two filtering mechanisms, we must be aware of the different technologies used for comparisons. Perhaps the LFE option is most accurate in measuring steps taken during the free-living environment and more telling for actual time spent in the different physical activity categories. Nonetheless, until studies are conducted to validate steps in the free-living environment, these authors are unable to determine which filtering option is most accurate.

Although this study has novel findings, it is not without limitations. First, our findings should not be extrapolated to the general African-American population considering the cross-sectional nature of the study and its small, homogeneous sample. Second, physical activity in the current participants was monitored in a free-living environment. As such, investigators must assume participants wore the monitors as instructed in order to obtain the most accurate physical activity measurements. To encourage general wear and minimize wear variation, monitors were placed on a belt and participants were given instructions on how to put on and take off the belt retaining the monitors at the beginning and end of each day. Time on and time off data were recorded by each participant to determine overall compliance and ensure all measurements included the majority of the individual’s daily activity. Finally, even though the current study did not utilize a criterion method as used by other investigators in previous studies [25], the devices have been shown to be valid tools for measuring physical activity [9, 26, 27].

## Conclusion

These data suggest that measuring and analyzing physical activity using the AG-LFE, compared to the AG-N and NL-800, will estimate higher daily steps among inactive individuals in a free-living environment. However, at this time, and based on the available data, it is unclear which filtering mechanism is most accurate in measuring step-counts and which cut points should be used to determine physical activity classifications. Additional research should be conducted on both the function of the LFE and its accuracy in this population and others, to further elucidate the effects of the LFE filtering mechanism on step counts and physical activity patterns. In addition, it will be important for researcher to develop appropriate cut-points to determine sedentary, light and MVPA cut-points using the LFE to obtain a more accurate assessment of physical activity in the free-living environment for young men and women.

## Supporting information

S1 File Minimal data set file. This file contains unidentifiable descriptive and activity monitor data for all study participants.
(XLSX)
